# Life table construction for crapemyrtle bark scale (*Acanthococcus lagerstroemiae*): the effect of different plant nutrient conditions on insect performance

**DOI:** 10.1038/s41598-022-15519-6

**Published:** 2022-07-06

**Authors:** Runshi Xie, Bin Wu, Mengmeng Gu, Hongmin Qin

**Affiliations:** 1 Department of Horticultural Sciences, Texas A&M University, College Station, TX 77843 USA; 2 Department of Horticultural Sciences, Texas A&M AgriLife Extension Service, College Station, TX 77843 USA; 3 Department of Biology, Texas A&M University, College Station, TX 77843 USA

**Keywords:** Invasive species, Entomology, Population dynamics, Plant sciences, Abiotic, Herbivory

## Abstract

Crapemyrtle Bark Scale (*Acanthococcus lagerstroemiae*; CMBS) is an invasive pest species that primarily infest crapemyrtles (*Lagerstroemia spp.*) in the United States. Recent reports have revealed the dire threat of CMBS to attack not only crapemrytles but also the U.S. native species with expanded host plants such as American beautyberry (*Callicarpa spp.*) and *Hypericum kalmianum* L. (St. Johnswort). A better understanding of plant–insect interaction will provide better and environmental-friendly pest management strategies. In this study, we constructed the first comprehensive life table for CMBS to characterize its biological parameters, including developmental stages, reproductive behavior, and fecundity. The indirect effects of three plant nutrient conditions (water, 0.01MS, and 0.1MS) on CMBS populations were examined using the age-stage, two-sex life table. The demographic analyses revealed that the plant nutrient conditions had significantly altered CMBS development in terms of the intrinsic rate of increase (*r*), the finite rate of increase (*λ*), the net reproductive rate (*R*_*0*_), and mean generation time (*T*). Higher *r*, *λ*, and *R*_*0*_ were recorded under nutrient-deficient conditions (water), while CMBS reared on plants with healthier growing conditions (0.1MS) had the most prolonged *T*. Overall, CMBS shows better insect performance when reared on plants under nutrient-deficient conditions.

## Introduction

Crapemyrtle (*Lagerstroemia* spp.), which generates an increasing market value of up to $70 million per year^1,2^, is a versatile ornamental plant widely used in landscape applications. Crapemytle is relatively easy to maintain in the landscape without severe disease and insect complications^3^. However, the infestation of crapemyrtle bark scale (CMBS; *Acanthococcus lagerstroemiae*) is spreading in the southeastern United States^4^, which dramatically affects the aesthetic and economic value of crapemyrtle^5^.

Crapemyrtle bark scale is a hemipterous insect under the superfamily of Coccoidea (scale insects), which is closely related to other piercing-sucking insects such as mealybugs (Pseudococcidae), aphids (Aphididae), whiteflies (Aleyrodidae), and psyllids (Psyllidae)^6^. According to previous studies and observations, the number of CMBS generations within one-year ranges from two to four depending on the climate zones^5,7,8^. In the Southeastern United States, CMBS-infested plants can be found in the field year-round, with up to four generations of CMBS being observed in Dallas, TX in 1 year^5^. However, the lack of in-depth characterization of CMBS biological parameters, such as developmental stages, reproductive behavior, and fecundity, limits the research capacity to investigate controlling strategies based on insect biology and physiology. The construction of life tables could contribute fundamental knowledge and provide insight into the population dynamics of a specific arthropod of interest. Hence, life table study and analysis would be essential tools to generate a holistic view of the basic biological properties in a CMBS population.

Initially used for human demographic studies, life tables comprise a set of parameters to represent the age-specific survival or mortality rates of different populations. The life table study has been adapted as a record-keeping system and mathematical approach to collect and interpret insect population dynamics^9^. Traditionally, methods proposed by Leslie^10^ or Birch^11^ have been widely used to construct age-specific life tables that focus on female individuals in a population^12–14^. For example, female age- or stage-specific life tables were constructed for phloem-feeding insects such as aphids^15–17^, armor scale insects^18–20^, soft scale^21,22^, mealybug^23^, and psyllid^24^. However, the life table construction solely based on females may lead to errors in calculating the population parameters, especially when bisexual arthropods are involved^14^. The traditional female life table also fails to distinguish developmental stages and accurately present the stage overlapping^12,25^. To address these issues, Chi and Liu^13^ developed the age-stage, two-sex life table analysis, which accounts for both sexes and developmental stages to achieve a comprehensive understanding of insect biology and ecology. In recent years, age-stage, two-sex life tables have been increasingly used for evaluating the performance of phloem-feeding insects such as aphids^26–29^ and armor scale insects^30^. However, a comprehensive life table study for CMBS has not been reported yet. Previous studies on CMBS tend to ignore the developmental stages due to the small size of the nymphs (0.3–3 mm depending on the age and sex)^31^. The technical difficulties in distinguishing immature nymphal stages remain challenging for constructing comprehensive life tables for CMBS.

A comprehensive life study promotes a better understanding of the plant–insect interactions under various conditions. Insects are ectothermic organisms. Therefore, environmental factors such as temperature and humidity greatly affect insect performance^32–34^. On the other hand, these abiotic factors combining plant nutrient conditions could indirectly affect insect herbivores by changing the host plants’ metabolic state. The study of abiotic factors by tracking individual CMBS in the field has been proven difficult, partially due to the relatively small size and the lifestyle of CMBS (feeding at cracks and crevices of stem or trunk). Therefore, a controlled laboratory condition with the aid of a microscope is necessary to conduct a comprehensive life study and investigate plant–CMBS interactions by excluding certain environmental variables.

Abiotic factors such as climate, light, and edaphic factors affect plant growth by altering plant metabolism, indirectly affecting insect herbivores. Hence, abiotic factors’ direct or indirect effects on insects are often important subjects of insect ecology. For example, as a major source of plant nutrients, edaphic environments alter plant physiology in terms of phenology and chemical property in plant tissues, which further affect the plant–insect interactions^35,36^. Plant malnutrition was known to disturb plant metabolism, which leads to nutrient-deficiency symptoms, including stunted growth, chlorosis, and necrosis of leaves^37^. Moreover, host plants show varied susceptibility towards the same pest species grown under different edaphic conditions^38^. Such plant–insect interactions associated with plant nutrition for CMBS are currently less understood. Therefore, we have investigated the CMBS life table by artificially altering the nutrients available to the host plants in this study.

Developing effective integrated pest management (IPM) programs is essential for predicting and preventing damages from CMBS in the United States. To reach this goal, we have conducted this study with two main objectives: (1) to construct the first comprehensive life table and gain a thorough understanding of CMBS biology in terms of its developmental stages, survival, and fecundity on *Lagerstroemia*; (2) to expand the current knowledge on the effects of plant nutrient conditions on the interaction between CMBS and its host plants, through the age-stage, two-sex life table analysis.

## Results

### Crapemyrtle bark scale life history

The CMBS rearing experiments were conducted using small, rooted plants grown inside small petri dishes (feeding chambers; Fig. S1). Daily monitoring was conducted under a dissecting microscope (see “Methods” section for details), which allows detailed documentation and characterization of all developmental stages of CMBS in its entire life cycle. According to our observation, the life cycle of CMBS reared on the seedling (cuttings) of *L. fauriei* ‘Fantasy’ under laboratory conditions varied between 102 and 158 days. The egg incubation time for males and females was 10.84 ± 0.05 (mean ± SEM, n = 396) days under 25 °C. The life history, including all developmental stages and significant events in the life cycle of CMBS, has been summarized in Fig. S2. The developmental stages for individual insects, such as the 1st and 2nd instars, can be determined by monitoring molting events. Since the molting process (last around 3–4 h; Supplementary Video 1) is relatively short compared to the entire life cycle, it was rarely captured live. Hence, the determination of stage changes was more easily facilitated by keeping track of the insect exuviae. Out of the three nutrient conditions evaluated, the ‘water’ or nutrient deficient treatment group shows utmost insect performance. Hence, the life history of CMBS males and females under ‘water’ condition has been characterized as below.

**Figure 1:**
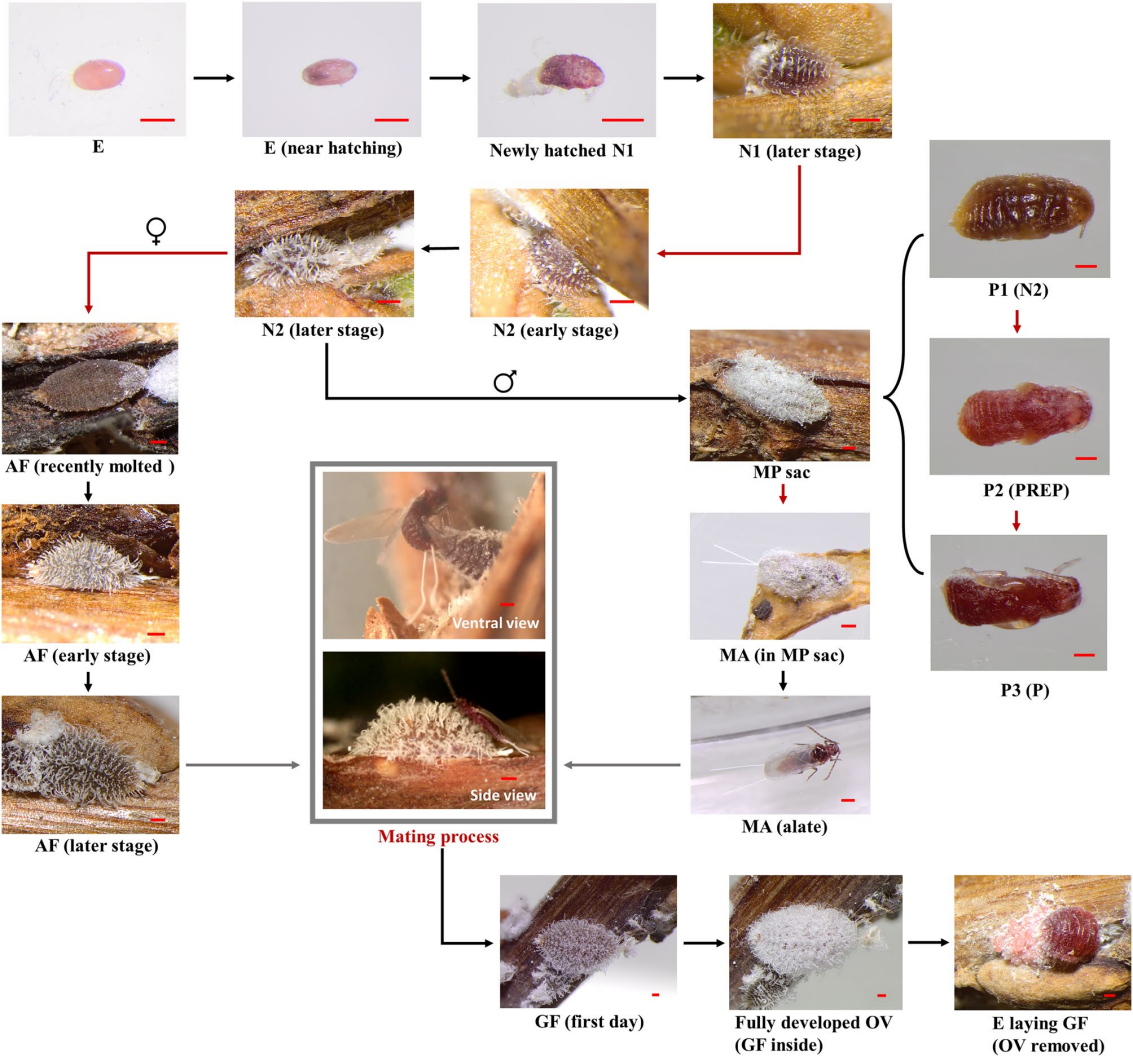
Detailed life history of crapemyrtle bark scale, *Acanthococcus lagerstroemiae*. E: Egg, N1: first-instar nymph, N2: second-instar nymph (N1 and N2 are indistinguishable in male and female), AF: adult female (or female third-instar nymph), MP sac: male pupa sac (consist of three stages: P1, P2, and P3), PREP: prepupa, P: pupa, GF: gravid female, OV: ovisac. Scale bars: 200 μm. Arrows represent chronological progression. Red arrows: molting events. Gray arrows and outline: adult males emerge and locate sessile adult females to perform the mating process.

The life history of CMBS males started from egg, followed by 1st instar [17.9 ± 0.6 (mean ± SEM, n = 17) days] and then 2nd instar [63.9 ± 5.0 (mean ± SEM, n = 17) days]. The development of the male pupa was characterized as a three-step process (P1, P2, and P3 stages). Firstly, a P1 stage has been identified, as a 2nd instar forms the elongated ‘cocoon’ structure typically identified as a male sac in the field (Fig. 1). Before P1, 2nd instar nymphs typically retract and detach their stylets from the plant and become mobile (Supplementary Video 2). Within a short period (24 h), the male 2nd instar will relocate until finally settle down at a location, then start excreting wax to form the white male sac (Supplementary Video 3). After P1, the 2nd instar will pupate and push out the exuviates through the rear opening of the male sac (Supplementary Video 4). The duration of P1 is around 4.6 ± 0.4 (mean ± SEM, n = 15) days, followed by prepupa (P2) and then pupa (P3), which takes 5.7 ± 1.5 (mean ± SEM, n = 12) days and 7.2 ± 0.7 (mean ± SEM, n = 12) days, respectively. Hence, a total of three exuviates will be pushed out from the male sac before adult male emergence. After the molting of the pupa, the adult male usually stays in the pupa sac for several days before emergence. Once exiting the pupa sac, the adult male immediately roams the surrounding environment, presumably searching for the adult female’s presence. The development of CMBS males is complete metamorphosis, as pupa and adult male stages can be identified (Fig. 1).

Compared to males, the female scale development undergoes incomplete metamorphosis, in which four major stages (egg, 1st instar, 2nd instar, and adult female) without pupation were recorded (Fig. 1). The duration for 1st and 2nd instar females is 18.2 ± 1.0 (mean ± SEM, n = 13) days and 61.4 ± 3.7 (mean ± SEM, n = 13) days, respectively. The adult (or the 3rd instar) female could be distinguished from the 2nd instar morphologically. The adult female gains size quickly and have a significant amount of wax secretion/development than the 2nd instar. The longest developmental stage for both males and females is the ‘2nd instar’ stage. The sex of CMBS could only be identified morphologically under live monitoring conditions after the 2nd instar due to the lack of significant difference among the 1st or 2nd instars regarding sex development.

### Mating behavior

Mating behavior was observed between adult males and adult females, which is an essential process required for reproduction. No parthenogenesis was observed under the experimental conditions in this study. Newly emerged adult females typically remain mobile for a short period before settling down at a suitable location to feed on the plants. Thus, most adult females become sessile for the rest of the life cycle, while the alate adult male (once located the female) would initiate the mating process by taping the dorsal side of the female. Upon stimulation, the female reacts by lifting and retracting the rear end of her abdomen to accept copulation (Supplementary Video 5). The male then proceeds to curve its abdomen down and direct its genitalia to contact the ventral side of the female abdomen, where sperm transfer could be occurring (Fig. 1; Supplementary Video 6).

After the mating process is completed, a reproductive female can be confirmed as the female develops its typical white ovisac structure (Fig. 1). As a newly emerged adult female gaining its size, it might be undergoing development for sexual maturity as various adult preproduction periods were recorded. Therefore, depending on the female ages, it would take from 2 to 11 days before the development of ovisac could be observed. Shortly after the ovisac is developed (2–3 days), a female would start laying eggs, and the reproduction period can last up to 10 days.

### Developmental duration and longevity

Murashige and Skoog (MS) basal salt, as a well-balanced nutrient source, is widely used in plant propagation applications such as tissue culture. Hence, the different levels of MS supplementation in media have led to apparent differentiation of plant development in terms of physical characteristics and potentially plant metabolism. Plants grown in nutrient-deficient media (the ‘water’ treatment) demonstrated the characteristics of plant malnutrition development, including stunted growth and yellowish leaves, compared to the plants under sufficient nutrient treatment (Fig. S1), which indirectly affects the development of CMBS.

Developmental duration, adult longevity, and total longevity of CMBS reared on *Lagerstroemia* host plants (with the same genetic background) under different nutrient conditions are listed in Table 1. The durations of immature nymphs (1st and 2nd instars) and the preadult duration were by large not affected by plant nutrient conditions, especially between ‘water’ and ‘0.01 MS’, while the comparison is more meaningful regarding the survival rate since the individuals under ‘0.1 MS’ treatment had significantly higher mortality than the other two treatment groups. Only one male and two females in the ‘0.1 MS’ group reached the respective adult stages. The adult longevity for all males was two to three days, while all adult females lived around one month, with no significant difference (*P* > 0.05) under three nutrient conditions (Table 1).

**Table 1:** Developmental duration, adult longevity, and total longevity of *Acanthococcus lagerstroemiae* reared on *Lagerstroemia* under different nutrient conditions.

Biological parameters	Stage	Water	0.01MS	0.1MS
		Mean ± SE		
Developmental times (days)	1st instar	18.31 ± 0.41a	17.23 ± 0.56 a	18.03 ± 0.48 a
	2nd instar + Pupa (male)	83.18 ± 6.59 a	91.13 ± 6.34 a	56.00 ± 0.00 b
	2nd instar (female)	62.09 ± 4.37 a	70.22 ± 6.50 a	69.5 ± 4.00 a
	Preadult stage	102.45 ± 4.56 a	107.24 ± 5.16 a	95.33 ± 3.59 a
Adult longevity (days)	Male	3.18 ± 0.30 ab	2.38 ± 0.32 b	3.00 ± 0.00 a
	Female	33.64 ± 4.58 a	21.67 ± 4.56 a	22.00 ± 10.14 a
Total longevity (days)		88.22 ± 5.23 a	89.69 ± 5.90 a	51.47 ± 3.77 b

Values are means ± standard errors; data within a row followed by different letters are significantly different at *p* < 0.05 by using paired bootstrap test.

Despite the lack of difference in the developmental times and longevity of the individuals that completed all life stages, the total longevity of all individuals (including nymphs who died immaturely) was vastly different between deficient and sufficient nutrient conditions. The total longevity of CMBS under ‘water’ (88.22 ± 5.23 days) and ‘0.01MS’ (89.69 ± 5.90 days) was almost double of the ones under ‘0.1MS’ (51.47 ± 3.77 days) (*P*_*water&0.01MS*_ = 0.85; *P*_*water&0.1MS*_ < 0.001; *P*_*0.01MS&0.1MS*_ < 0.001) (Table 1).

### Fecundity and oviposition days

Crapemyrtle bark scale females had similar fecundity in terms of adult preoviposition period (APOP), total preoviposition period (TPOP) fecundity, and oviposition days in the ‘water’ and ‘0.01MS’ groups (*P* > 0.05; Table 2). Compared to ‘0.01MS’, female CMBS under ‘0.1MS’ had longer APOP and TPOP (*P* < 0.05). The highest fecundity observed was 102 eggs from one female in the ‘water’ group, while the lowest fecundity was 48 eggs from one female in the ‘0.1MS’ group.

**Table 2:** Adult preoviposition period (APOP), total preoviposition period (TPOP), fecundity, and oviposition days of *Acanthococcus lagerstroemiae* reared on *Lagerstroemia* under different nutrient conditions.

	Water	0.01MS	0.1MS
	Mean ± SE		
APOP (days)	30.14 ± 5.19 ab	22.80 ± 2.31 b	35 .00 ± 0.00 a
TPOP (days)	125.47 ± 7.15 b	118.80 ± 7.73 b	140.00 ± 0.00 a
Fecundity (eggs)	49.18 ± 12.40 a	34.22 ± 10.69 a	24.00 ± 13.51 a
Oviposition days	8.86 ± 0.41 a	8.4 ± 0.25 a	5.00 ± 0.00 b

Values are means ± standard errors; data within a row followed by different letters are significantly different at *p* < 0.05 by using paired bootstrap test.

### Life table analysis of CMBS

For life table/population parameters, no significant difference was detected between ‘water’ and ‘0.01MS’ (*P *> 0.05). However, CMBS population under both ‘water’ and ‘0.01MS’ had higher growth rate in terms of intrinsic rate of increase (*r*) (*P*_*water&0.01MS*_ = 0.71; *P*_*water&0.1MS*_ = 0.0014; *P*_*0.01MS&0.1MS*_ = 0.0048) and finite rate of increase (*λ*) (*P*_*water&0.01MS*_ = 0.72; *P*_*water&0.1MS*_ = 0.0016; *P*_*0.01MS&0.1MS*_ = 0.0073) compared to CMBS reared under ‘0.1MS’ (Table 3). The highest *r* and* λ* among the three treatments were 0.018 ± 0.003/days and 1.018 ± 0.023/days, respectively, on ‘water’. Meanwhile, *r* and* λ* were − 0.0018 ± 0.002/days and 0.998 ± 0.0482/days, respectively, from ‘0.1MS’ (Table 3). According to the life table theory, an *r* value of less than 0 or *λ* less than 1 would lead to a negative value in the doubling time, suggesting the decline of population size^39^. The longest mean generation time (*T*) was 142.86 ± 1.92 days on plants under the ‘0.1MS’ condition, compared to the shortest of 121.37 ± 7.53 days on ‘0.01MS’ (Table 3).

**Table 3:** Population parameters, including the intrinsic rate of increase (*r*), finite rate of increase (*λ*), net reproduction rate (*Ro*), mean generation time (*T*), and gross reproductive rate (GRR) of *Acanthococcus lagerstroemiae* under different nutrient conditions.

Conditions	*r* (/days)	*λ*	*Ro*	*T* (days)	GRR (offspring/individual)
Water	0.018 ± 0.003 a	1.018 ± 0.023 a	9.84 ± 3.57 a	127.35 ± 7.51 b	95.51 ± 31.55 a
0.01MS	0.017 ± 0.004 a	1.017 ± 0.075 a	7.33 ± 3.10 a	121.37 ± 7.53 b	61.95 ± 27.35 a
0.1MS	− 0.0018 ± 0.002 b	0.998 ± 0.482 b	0.77 ± 0.77 b	142.86 ± 1.92 a	48.00 ± 0.00 a

Values are means ± standard errors; data within a column followed by different letters are significantly different at *p* < 0.05 by using paired bootstrap test.

**Figure 2:**
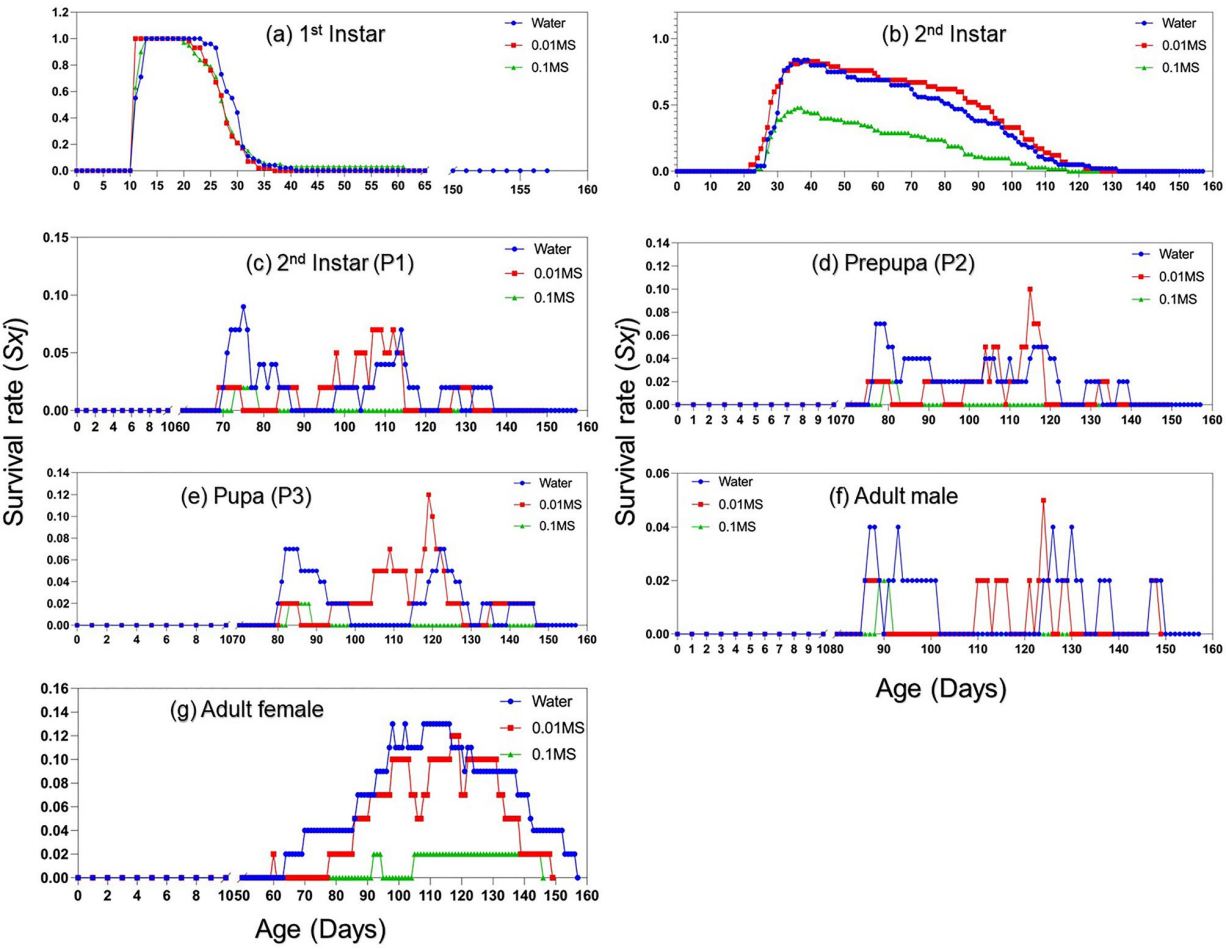
Age-stage specific survival rate (*s*_*xj*_) of *A. lagerstroemiae* at different nutrient conditions (Water, 0.01MS, and 0.1MS), grouped by different stages: (**a**) 1st instar, (**b**) 2nd instar, (**c**) 2nd instar (P1), (**d**) prepupa (P2), (**e**) pupa (P3), (**f**) adult male, and (**g**) adult female.

The age-stage specific survival rate (*s*_*xj*_) of CMBS reared under different host conditions provides various probabilities that a newborn within a particular cohort (treatment group) would survive to age *x* and stage *j* (Fig. 2). The overlaps of survival curves resulted from the variable developmental rate of individuals at different life stages, which were observed under all three nutrient conditions (Fig. S3). The development of CMBS under three nutrient conditions resembles each other at the beginning of the rearing experiment, as the survival curves of 1st instar followed a similar pattern. The emergence of the 2nd instar started 20 days after the rearing experiment and reached peaks at around 30–40 days, where a significant difference in survivor rates was recorded. The survival rate of the 2nd instars under ‘water’ and ‘0.01MS’ was almost double compared to that of the ‘0.1 MS’ group (Fig. 2b). This phenomenon resulted from the higher mortality of 1st and 2nd instar under ‘0.1 MS’ in the first 60 days, leading to fewer individuals reaching the later stages, including 2nd instar (P1), prepupa (P2), pupa (P3), adult male, and adult female (Fig. 2).

**Figure 3:**
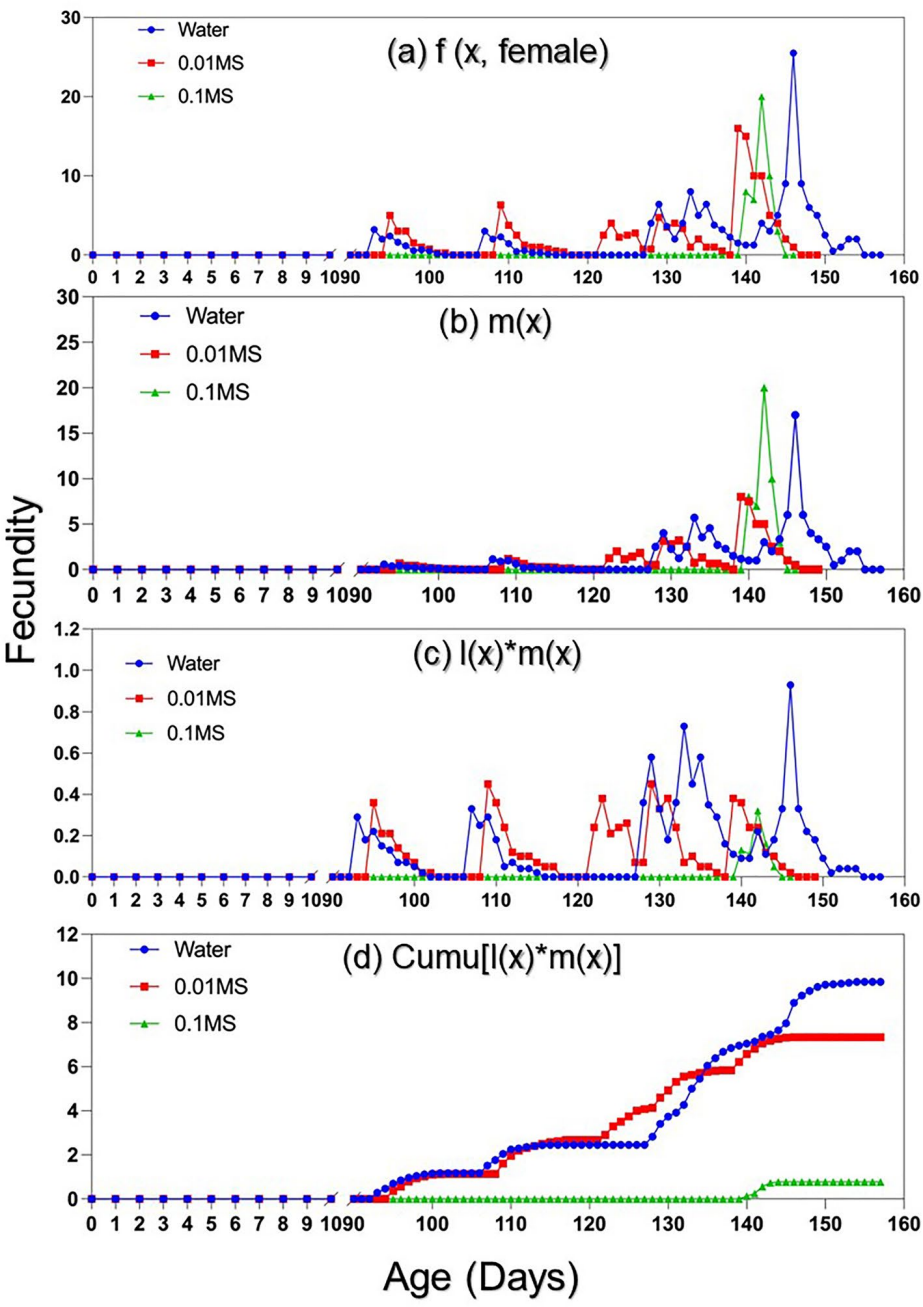
Fecundity of *Acanthococcus lagerstroemiae*, including (**a**) age-stage specific fecundity [*f* (*x*, female)], (**b**) age-specific fecundity (*m*_*x*_), (**c**) age-specific maternity (*l*_*x*_*m*_*x*_), and (**d**) cumulative net reproduction rate (*R*_*x*_) at different nutrient conditions (Water, 0.01MS, and 0.1MS).

The age-stage specific fecundities [*f* (x, female)], representing the mean number of eggs produced by females at age *x*, were reported in Fig. 3. The maximum daily fecundity [*f* (x, female)] was 25, 16, and 20 eggs for ‘water’, ‘0.01MS’, and ‘0.1MS’, respectively. While the maximum daily fecundity was achieved between day 140 and day 150 for all three treatments, the females under ‘water’ and ‘0.01MS’ started reproducing much earlier than ‘0.1MS’. For example, the population under ‘water’ generated fecundity data as early as day 93 compared to day 140 for individuals under the ‘0.1MS’ condition (Fig. 3)*.* The curves for the age-specific fecundity (*m*_*x*_), age-specific maternity (*l*_*x*_*m*_*x*_), and the cumulative reproductive rate (*R*_*x*_) were also provided in Fig. 3. The *l*_*x*_*m*_*x*_ and *R*_*x*_ under ‘0.1MS’ were lower than ‘water’ or ‘0.01MS’ (Fig. 3). This is due to the *l*_*x*_, which gives the probability that an egg will survive to age *x*, was very low for individuals under ‘0.1MS’.

**Figure 4:**
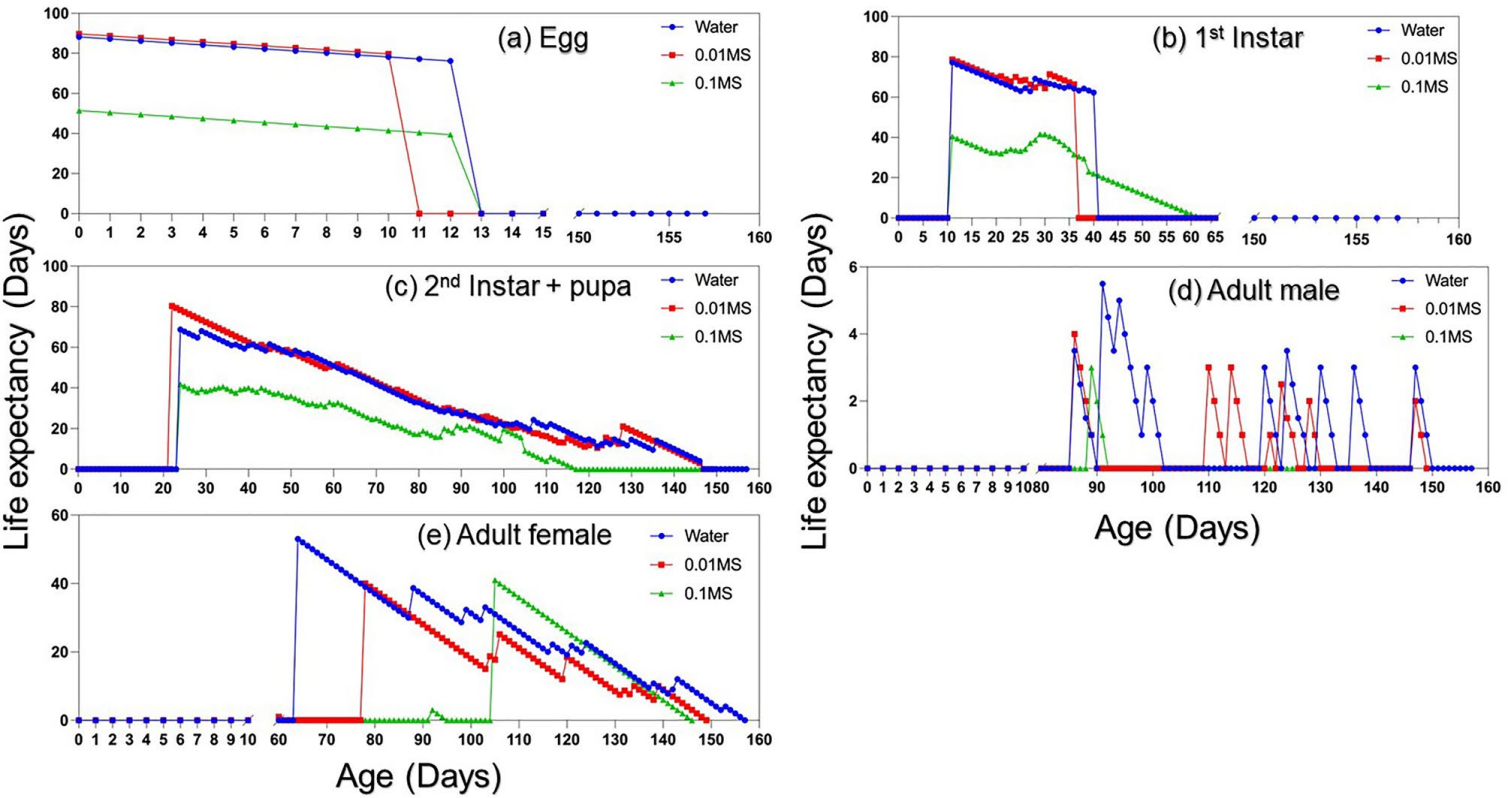
Age-stage specific life expectancy (*s*_*xj*_) of *Acanthococcus lagerstroemiae* at different nutrient conditions (Water, 0.01MS, and 0.1MS), grouped by different stages: (**a**) egg, (**b**) 1st instar, (**c**) 2nd instar + pupa stages, (**d**) adult male, and (**e**) adult female.

The age-stage specific life expectancy (*e*_*xj*_) reports the duration that an individual of age *x* and stage *j* would be expected to live^40^. The maximum life expectancy was observed under ‘water’ while the lowest was recorded for individuals living under ‘0.1MS’ (Fig. 4).

## Discussion

As a newly emerged non-native pest species, many aspects of CMBS biology and its potential adaptive development in the United States remained unknown. This study characterized the CMBS developmental stages and life cycle in great detail through a comprehensive life table study since no such report was available, especially under controlled laboratory conditions. Most previous research on CMBS relied on observation or experiments in the field, such as monitoring programs of crawler activity^41^ and tests on chemical efficacy^42–44^, which provides valuable experience in controlling the CMBS infestation. However, the application of pesticides exposes humans and beneficial insects to potential hazards, which may lead to unbalanced biodiversity^45^. Furthermore, the development of insecticide-resistant scale populations has been reported as the direct consequence of long-term overuse of insecticide^46^. Therefore, environmental-friendly pest management programs with ecological-oriented strategies, such as biological control^47^ or mating disruption^48–52^, should be developed for CMBS control to avoid or reduce pesticide usage. The development of these environmental-friendly strategies requires a deeper understanding of insect biology, including life cycle, population dynamics, and mating behavior.

A better understanding of the CMBS biology and the developmental process has been achieved, for the first time, through the age-stage, two-sex life table construction in this study. The CMBS rearing system was essential and efficient for rearing and tracking individual CMBS nymphs, allowing detailed observations to generate the life history data in this study. According to our observation in the CMBS rearing experiment, the life history of a male involves six major stages: egg, 1st instar, 2nd instar, P1 (2nd instar male after forming pupa covering), P2 (prepupa), P3 (pupa), and an adult alate stage. Four developmental stages were recorded in females: egg, 1st instar, 2nd instar, and an adult stage (Fig. 1). Previously, incongruent reports on CMBS life history were found in the literature. For example, Zhang and Shi reported two instars before females become sexually mature^53^, while Jiang and Xu reported both a 3^rd^ instar and an adult stage in female development^7^. Our results were consistent with Zhang and Shi^53^, who reported five stages for males and three for females (excluding the egg stage).

The second objective of this study is to identify the effects of plant nutrient conditions as a factor that can influence plant physiological conditions and the plant–insect interactions. Both abiotic and biotic factors are integrated elements that regulate the population dynamics and distribution of insect herbivores^54,55^. Abiotic factors refer to non-living components such as temperature, humidity, light, and minerals^54^, while biotic factors are elements related to living organisms such as natural enemies^56^ in an ecosystem. The plant–herbivore interactions are crucial in understanding insect biology and developing an effective and eco-friendly integrated pest management program^57^.

Hence, we have investigated the indirect effects of different host conditions, under three different nutrient conditions, on the biological parameters of CMBS in this study. We utilized the supplementation of Murashige and Skoog (MS) basal salt without vitamins to create different plant nutrient conditions. As a universal standard medium formulation, the MS basal salt provides a complete and well-balanced list of required micro-and macro-nutrients for plant growth^58^. As a result, the rooted clones grown in three different media started to show different growth conditions after one month. Stem cuttings grown in nutrient deficient water agar medium had a noticeable slower shoot and leaf growth and yellowish leaf compared to the healthiest plant growth in the ‘0.1MS’ agar medium (Fig. S1). However, better plant growth conditions did not promote CMBS infestation. Instead, *R*_*0*_ was less than 1 when under ‘0.1MS’, suggesting that the CMBS population could not increase and would eventually die out under this condition.

With an initial intention to optimize CMBS colonies using healthy host plants, this result was somehow unexpected and contradictory to some reports on other hemipteran species. Previous studies have shown that applying nitrogen fertilizers to soil leads to the increased severity of mealybugs infestation in terms of enhanced fecundity and shortened insect generation time^59^. Phosphorus did not affect the mealybugs population, whereas increased potassium level in the medium results in decreased infestation^60^. The elongate hemlock scale (*Fiorina externa* Ferris) showed better performance with lower mortality and higher fecundity on healthy plants than plants under adverse growing conditions^61^. Interestingly, different species of scale insects might respond differently to the availability of macro- or micro-nutrients in soil. Salama et al. reported in 1972 that the supplementation of nitrogen, potassium, and phosphorous reduced the susceptibility of citrus seedlings to the infestation of the red scale, *Aonidiella aurantii*^62^. The effect of plant nutrition on crapemyrtle metabolic pathway and its association with plant–CMBS interactions are currently less understood, and further research is needed.

The relationship between plant hosts and insects could be complicated, counter-intuitive, and subject to the ever-changing abiotic factors such as climate change^63^. For example, well-regulated abiotic factors in cropping systems, such as fertilization, are often desirable and have shown a positive influence on plant growth^64,65^ by optimizing nutrient uptakes^66^ and the synthesis of essential compounds^67^. However, better plant performance may or may not lead to increased plant defense against herbivore attacks. Insect fitness is determined by a combination of factors, including the nutritional quality and defensive property of host plants and the physiological conditions of insects^68^. For example, Sauge et al. showed that the infestation of green peach aphid (*Myzus persicae*) had a positive correlation with nitrogen fertilization up to a certain level, then became negative^69^. Chen et al. reported that the elevated carbon dioxide had negatively affected the performance of aphids feeding on Barley^70^.

In the case of scale insects, differentiated infestations on the same host plant species grown nearby^71^ are not uncommon. Similar phenomena have been observed with CMBS on the same plant species in field^5^ and across different plant species in host range tests^31^. The interaction between scale insects and their host plants has been studied extensively, and complicated research questions were proposed. For example, some scale insects show a certain level of specialty and feeding preference in infesting specific host plants, whereas some species were reported to be polyphagous^72^. As a significant source for providing plant nutrients, edaphic environments alter plant physiology in terms of phenology and chemical property in plant tissues, affecting plant–insect interactions^35,36^.

The underlying plant–insect interactions for CMBS have not been studied extensively, and hence related research is needed to develop effective and low-cost strategies for pest management. Therefore, the life table study is an effective tool that has been used extensively to investigate the effects of abiotic and biotic factors on insect development and plant–insect interactions^73–75^. The CMBS rearing systems developed in this study were proven successful in supporting the CMBS life cycle to better document and characterize the developmental stages of CMBS, including the defining differentiation and the duration of each developmental stage, which is essential for investigating the factors that affect insect mortality.

## Conclusion

In conclusion, we have constructed the first age-stage, two-sex life table for CMBS to better understand its biology and ecology. Crapemyrtle bark scale showed poorer performance in establishing a population on plants in healthier conditions. However, our current data should not conclude that a healthy crapemyrtle plant is immune to CMBS attack. The underlying plant defense mechanism, such as secondary metabolites under different nutrient conditions, is unknown and should be investigated in the future. Field experiments should further validate this phenomenon, in which more factors such as natural enemies might come into play. Therefore, future studies on other abiotic factors, such as temperature, humidity, and pesticides, should be investigated to optimize the understanding and insect management strategies.

## Methods

### Development of insect colony

Branches/twigs infested with crapemyrtle bark scale were collected from crapemyrtle trees on campus (Texas A&M University, College Station, TX) and stored in zip-lock bags under constant temperature (25 °C). Colonies of CMBS were developed by tying infested branches to healthy crapemyrtle plants. Plants inoculated with CMBS were then placed in plant/insect growth chambers. The plant/insect growth chambers were constructed with supporting frame material and mesh fabric. The plant/insect chambers containing the CMBS colonies were placed in a greenhouse or Conviron growth chamber (25 °C and 250 μmol/m^2^/s; 12:12 light:dark), which provides stable environmental conditions to allow long-term maintenance CMBS population. The gravid females used in this study were collected from laboratory-maintained insect colonies.

### Plant material and insect rearing chamber

Cuttings collected from one seedling of *Lagerstroemia fauriei* ‘Fantasy’ were used as host/food sources for the CMBS rearing experiment. The plant materials used for insect rearing were collected from a seedling, grown from one seed collected from a crapemyrtle plant (*Lagerstroemia fauriei* ‘Fantasy’). The original *L. fauriei* ‘Fantasy’ plant, from which the seeds were collected, was obtained with pessimism from the Department of Environmental Horticulture, University of Florida/IFAS North Florida Research and Education Center (Quincy, FL 32351, USA). A voucher specimen (accession no. TAES0367977) of the plant material used in this study was deposited in the S.M. Tracy Herbarium (TAES/TAMU) at Texas A&M University.

Stem cuttings were cleaned and soaked in 10% bleach solution for 10 min for sterilization. Cleaned cuttings were stuck in agar media for rooting. Murashige and Skoog (MS) basal salt (PhytoTech Labs, Lenexa, KS, USA) was utilized as a ‘complete’ supplementation of plant nutrients. Three different agar media were prepared and considered as three different treatments: water (nutrient-deficient condition), 0.01 MS (intermediate nutrient condition; 0.043 g MS salt per 1 L media), and 0.1 MS (nutrient-sufficient condition; 0.43 g MS salt per 1 L media). The rooted cuttings (clones) were used in the following rearing experiments. The rooted clones grown in three different media showed similar initial plant growth (initial sprouting and root development), but as time progressed, plants in nutrient-deficient medium showed noticeable plant malnutrition development (stunted growth and yellowish leaf) compared to the plants in nutrient-sufficient medium (Fig. S1).

Rearing chambers were constructed with small petri dishes (Falcon® Disposable Petri Dishes, 60 mm × 15 mm) and clear plastic food wrap. Around half of petri dish was wrapped by clear plastic food wrap to create space for the medium. Agar medium (1%) with different nutrient conditions was poured into the bottom of the petri dish, using an electronic pipette, to fill around one-third portion of the petri dish. Rooted stem cuttings with bud nodes were transferred from rooting containers to the insect rearing chambers before the experiments. Depending on the condition of the medium, rooted cuttings were carefully removed from the old medium and transferred to fresh medium regularly throughout the rearing experiment. The rearing chambers were placed in Conviron growth chambers set at 25 °C and 250 μmol/m^2^/s light with a photoperiod of 12:12 (light:dark).

### Insect rearing experiment

White coverings of the female scales were carefully lifted using a fine pin/needle. All existing eggs inside the ovisacs were removed, and the gravid females were transferred, using a fine brush, onto a moist filter paper placed in a petri dish. All newly laid eggs were collected the next day (after 24 h) and kept under 25 °C for incubation until hatched.

Egg incubation times were recorded, defined as the duration from the day when gravid females first laid the eggs to the day 1st instars were hatched. Five to ten newly hatched crawlers/nymphs per rearing chamber were transferred onto stem cuttings using a fine brush. Daily observations were made to record the settling status of nymphs. Rearing chambers with nymphs that failed to settle on the plants were discarded when the mortality or escaping were confirmed.

Insect rearing experiments on *Lagerstroemia* in different nutrient conditions were conducted from July to December 2019 and repeated from May to November 2020. Daily observations were conducted using a stereomicroscope as nymphs started feeding. Each individual within a feeding chamber was assigned an ID for tracking purposes. The duration of developmental stages (including nymphal stages, pupa, and adult stages) was recorded. When a male reaches the adult stage, it will be transferred to a pair with a female for mating to complete the female’s life cycle. Fecundity data (the number of eggs that an adult female produces) and longevity (the number of days a female lives) were recorded as the gravid females complete their life cycle. The life history data collected from all individual CMBS were pooled together for the life table analysis.

The entire developmental process of CMBS has been photographed using a stereomicroscope system (Olympus SZX7, Waltham MA, United States) coupled with a color camera (Olympus LC30, Waltham MA, United States). All photos were generated by manual focusing along the z-axis, and then focus stacking was performed using Helicon Focus (Helicon Soft Ltd. Kharkiv, Ukraine).

### Data analysis

The developmental stages of both male and female CMBS were determined by the number of times the nymphs molt, which can be obtained most simply by keeping track of the exuviates. According to age-stage, two-sex life table theory^12,13^, the fecundity data (number of eggs each adult female produced) and longevity data (the number of days each CMBS nymph lives) can be obtained to calculate the population (life table) parameters of CMBS.

The life history data of CMBS were analyzed using TWOSEX-MS Chart, a computer program for the age-stage, two-sex life table analysis^76^, to obtain population (life table) parameters. According to the method described in Chi and Su^76^, the raw data were used to calculate the age-stage specific survival rate {*s*_*xj*_, where *x* = age in days and *j* = stage; the first stage is egg, the second stage is 1st instar, the third stage is 2nd instar, the fourth stage is male pupa 1 (2nd instar with cocoon), the fifth stage is male pupa 2 (prepupa), the sixth stage is pupa 3 (pupa), the seventh and eighth stages are female and male}, age-specific survival rate (*l*_*x*_), age-specific fecundity (*m*_*x*_), and population (life table) parameters, including mean generation time (*T*), net reproduction rate (*R*_*o*_), the intrinsic rate of increase (*r*), and the finite rate of natural increase (*λ*), to construct the age-stage, two-sex life table. In the age-stage, two-sex life table, the *l*_*x*_ and *m*_*x*_ are calculated as:

1 $${l}_{x}=\sum_{j=1}^{k}{s}_{xj}$$

and

2 $${m}_{x}=\frac{\sum_{j=1}^{k}{s}_{xj}{f}_{xj}}{\sum_{j=1}^{k}{s}_{xj}}$$

where *k* is the number of stages. *R*_*o*_, or the net reproduction rate, represents the mean number of eggs an individual CMBS produced during its entire life cycle, which is calculated as:

3 $${R}_{0}=\sum_{x=0}^{\infty }{l}_{x}{m}_{x}$$

*R*_*o*_ equals the sum of age-specific maternity (*l*_*x*_*m*_*x*_), which can be represented by the maximum of the cumulative reproductive rate, or *R*_*x*_ (formula shown below).

4 $${R}_{x}=\sum_{i=0}^{x}{l}_{i}{m}_{i}$$

*r*, or the intrinsic rate of increase, is calculated using the iterative bisection method with age indexed from 0^77^, and the formula is shown as:

5 $$\sum_{x=0}^{\infty }{e}^{-r\left(x+1\right)}{l}_{x}{m}_{x}=1$$

*λ*, or the finite rate of increase, is calculated as:

6 $$\lambda ={e}^{r}$$

*T*, or the mean generation time, represents the length of time (days) a population used to increase to *R*_*o*_-fold of its size and is calculated as:

7 $$T = \left( {\ln R_{o} } \right)/r$$

All data calculated for the biological parameters of CMBS were subjected to the bootstrap resampling with 100,000 replications to estimate the standard errors. Differences in the duration of each developmental stage and the population parameters of CMBS reared at different nutrient conditions were compared using the paired bootstrap test^78^.

### Statement of compliance

Experimental research on plants and collection of plant materials were performed in accordance with relevant institutional, national, and international guidelines and legislation.

## Supplementary Information

Supplementary Information 1.

Supplementary Video 1.

Supplementary Video 2.

Supplementary Video 3.

Supplementary Video 4.

Supplementary Video 5.

Supplementary Video 6.
