# Supplementary material for: Life table construction for crapemyrtle bark scale (Acanthococcus lagerstroemiae): the effect of different plant nutrient conditions on insect performance
Source: Sci Rep. 2022 Jul 6;12:11472. doi: 10.1038/s41598-022-15519-6 (PMC9259638; doi:10.1038/s41598-022-15519-6)
Supplement: Supplementary file 1 — Supplementary Information 1. [file 41598_2022_15519_MOESM1_ESM.docx]

# Life Table Construction for Crapemyrtle Bark Scale (*Acanthococcus lagerstroemiae*): the Effect of Different Plant Nutrient Conditions on Insect Performance

# Runshi Xie1,3, Bin Wu1,3, Mengmeng Gu2, *, and Hongmin Qin3, *

1Department of Horticultural Sciences, Texas A&M University, College Station, TX 77843, USA,

2Department of Horticultural Sciences, Texas A&M AgriLife Extension Service, College Station, TX 77843,

3Department of Biology, Texas A&M University, College Station, TX 77843.

*Mengmeng.Gu@ag.tamu.edu

*hqin@bio.tamu.edu


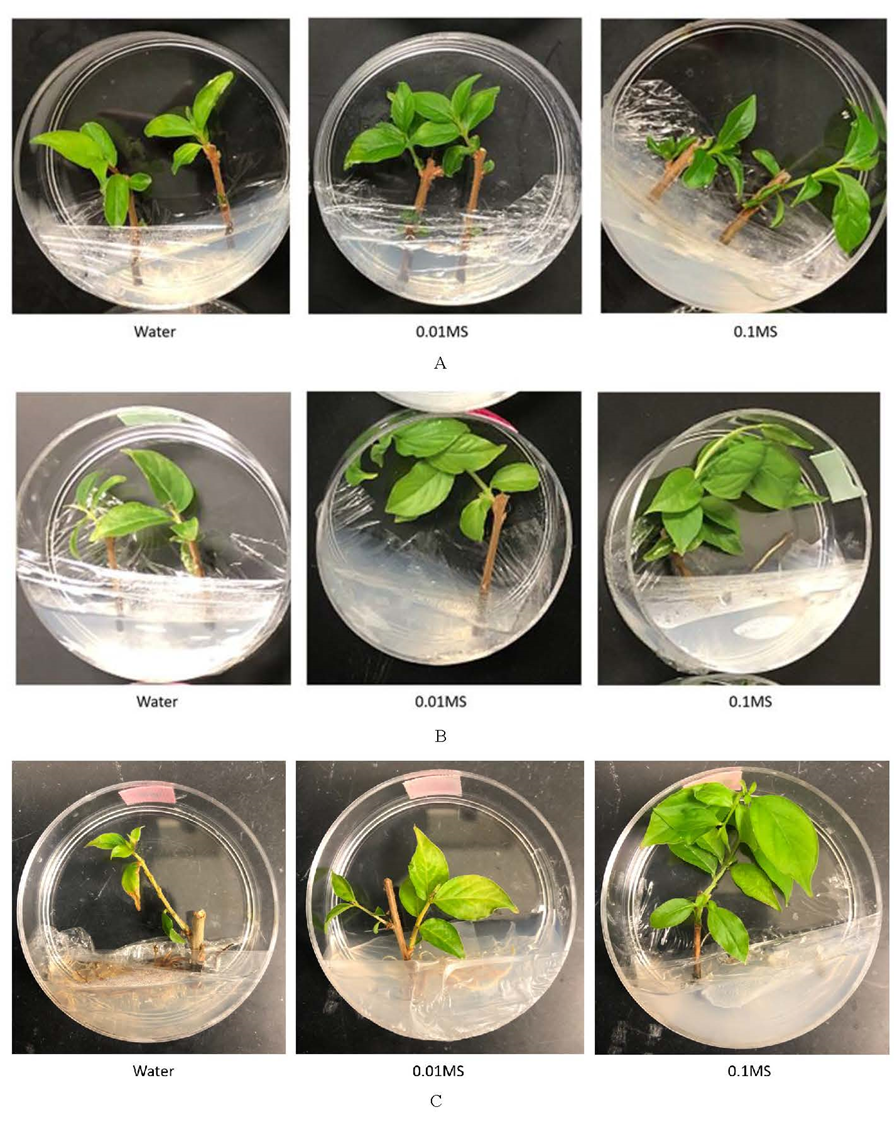


**Figure S1.** Rooted cuttings of one *Lagerstroemia fauriei* ‘Fantasy’ seedling growing under three different nutrient conditions (Water, 0.01MS, and 0.1MS) in four months (prior to insect inoculation). A: one month after planting (MAP); B: two MAP; C: four MAP.

*
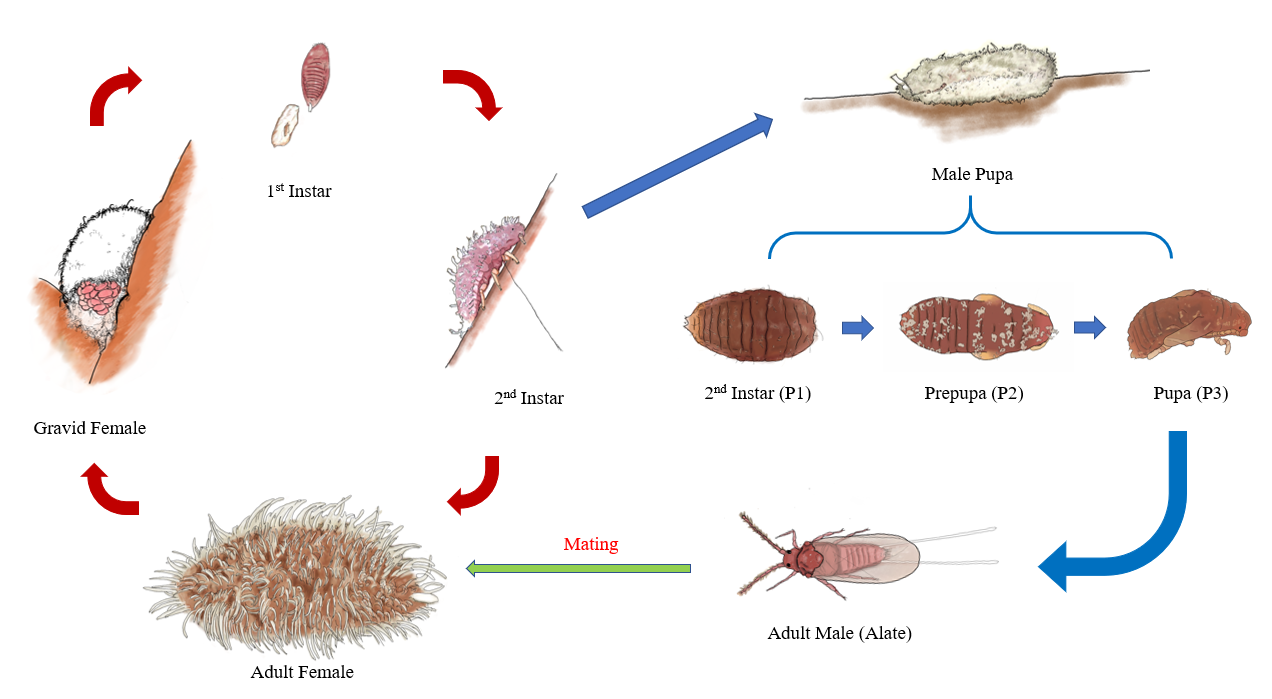
*

**Figure S2.** Life cycle (summary) of crapemyrtle bark scale, *Acanthococcus lagerstroemiae.*


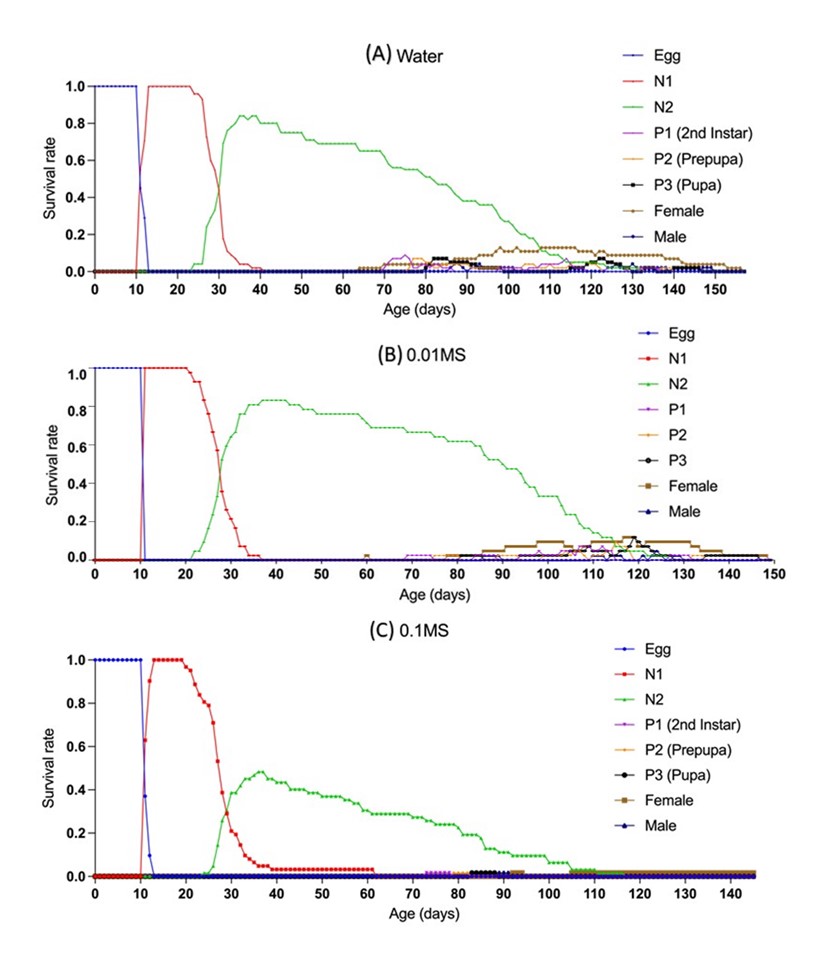


**Figure S3.** Age-stage specific survival rate (*s_xj_*) of *A. lagerstroemiae* at different nutrient conditions: (A) Water; (B) 0.01MS; (C) 0.1MS.


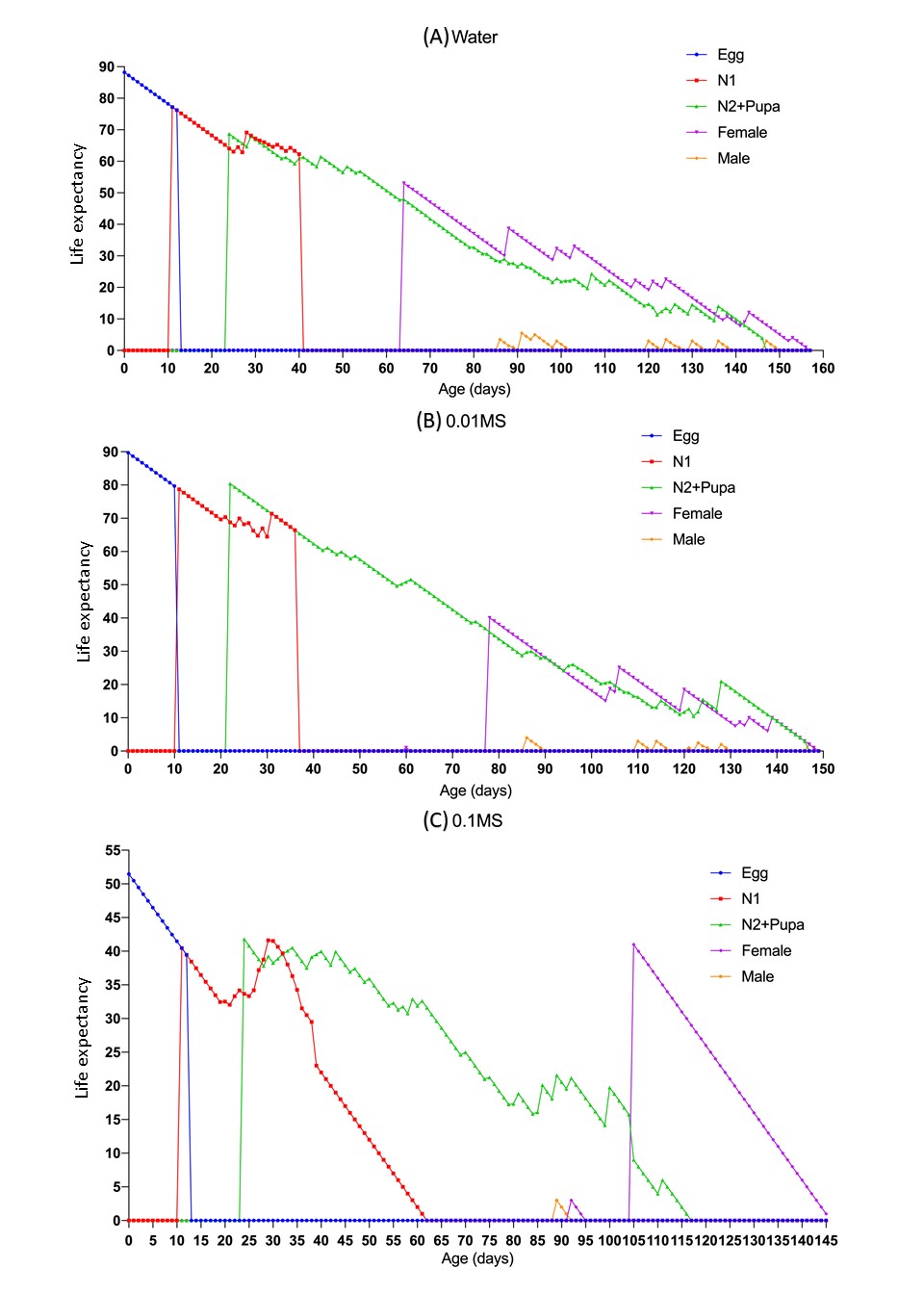


**Figure S4.** Age-stage specific life expectancy (*e_xj_*) of *Acanthococcus lagerstroemiae* at different nutrient conditions: (A) Water; (B) 0.01MS; (C) 0.1MS.

**Supplementary Video 1.** The molting process of a first instar (N1) *Acanthococcus lagerstroemiae*.

**Supplementary Video 2.** A second instar (N2) *Acanthococcus lagerstroemiae* is retracting its stylets and becoming mobile.

**Supplementary Video 3.** A second instar (N2) male *Acanthococcus lagerstroemiae* is forming the male sac.

**Supplementary Video 4.** A second instar *Acanthococcus lagerstroemiae* within the male sac (P1) is molting and pushing the exuviae out of the male sac.

**Supplementary Video 5.** The mating behavior of *Acanthococcus lagerstroemiae*: the female’s reactions upon stimulation by the male.

**Supplementary Video 6.** The mating behavior of *Acanthococcus lagerstroemiae*: copulation between male and female.
